# Acute effects of virtual-reality and dual-task warm-up on game-related speed paradigms in elite ice hockey athletes

**DOI:** 10.3389/fspor.2026.1766605

**Published:** 2026-03-25

**Authors:** Mark Brinkbäumer, Judith Ahle, Lukas Reichert, Marie-Therese Fleddermann, Karen Zentgraf

**Affiliations:** Department of Movement Science and Training in Sports, Institute of Sport Sciences, Goethe University, Frankfurt, Germany

**Keywords:** cognitive-motor interference, elite athletes, game speed, multitasking, sports performance

## Abstract

**Introduction:**

Dual-task (DT) performance is a key component of game speed in ice hockey, where athletes must maintain high skating velocities while processing perceptual and cognitive information. Although DT training (DTT) and virtual reality training (VRT) are increasingly used in high-performance settings, little is known about their acute effects on DT speed performance and individual variability in responsiveness. Therefore, this study examined (1) the acute effects of DTT and VRT warm-up on DT tapping speed with two different cognitive conditions and (2) related performance outcome measurements derived from cognitive testing and on-ice speed diagnostics.

**Methods:**

Seventy-four elite youth ice hockey players (17.3 ± 1.7 years) from U18 female, U18 male, and U20 male squads completed a simple tapping task (ST) and two DT paradigms - tapping paired with either a speed-reading task (SR) or a Stroop task (STR). Using a randomized crossover pre-post design, all athletes performed both a 20-minute DTT intervention and a 20-minute VRT intervention. Individual responsiveness was determined using the smallest worthwhile change (SWC). Moderating variables included cognitive test scores and on-ice speed performance.

**Results:**

Both DTT and VRT yielded significant improvements in ST performance (*p* < .001). DTT produced a significant improvement of DT tapping frequency with SR (*p* < .001), whereas VRT caused a small decline (i.e., lower frequencies). Neither intervention improved STR, and VRT was associated with more decrements than benefits across DT conditions. SWC analyses revealed notable interindividual variability: most athletes showed no certain change, but a subset demonstrated clear improvements or decrements. Correlation analyses indicated that DTT-induced improvements in STR were moderately associated with several on-ice measures (*ρ* = .27-.39, *p* < .05). No consistently related performance outcome measurements emerged for VRT. VRT did not lead to improved performance in DT paradigms requiring linguistic processing or inhibitory control and even produced motor performance decrements in some athletes.

**Discussion:**

Our study suggests that DTT appears more effective than VRT in acutely supporting DT speed performance, particularly in SR. The heterogeneity in responsiveness underscores the need for individualized cognitive-motor warm-up strategies and highlights the limitations of group-level outcomes.

## 1 Introduction

Ice hockey is among the fastest team sports globally in terms of player on-ice velocity, being characterized by numerous high-intensity skating bouts and changes of direction (COD), combined with frequent body impacts during offensive and defensive actions (1). Players rotate through brief, high-demand on-ice shifts throughout each period, creating a distinctly intermittent work-rest pattern (2–4). Bond et al. (2) note that a typical ice hockey match is very dynamic and involves exposure to intermittent high-intensity activity rather than continuous moderate work. The capacity to skate at high velocities appears to be one of the primary attributes of elite ice-hockey players (3). This observation aligns with evidence that athletes at higher competitive levels demonstrate superior maximal sprint performance compared to sub-elite counterparts (4). Enhanced speed and acceleration thus have the potential to improve match outcomes, as faster skaters traverse space more rapidly than opponents, thus potentially gaining positional and tactical advantage (3). A substantial body of research has examined methods to make team-sport athletes faster through strength, power, sprint or COD training (5–8). However, due to the multidimensional nature of ice hockey, game speed demands more than physical qualities. Specifically, ice hockey athletes still need to be able to keep up speed even when faced with complex, cognitively demanding game situations – an aspect addressed in the dual- and multitasking literature (9–11).

Koch et al. (12) define multitasking as the timely overlap of cognitive and motor processes when performing two (or more) tasks. Concurrent motor and cognitive tasks both require considerable cognitive resources, which can lead to cognitive-motor interference or decrease in one or both tasks. Even though athletes are regularly exposed to such scenarios, their performance seems to suffer under high load dual- or multitasking conditions [for a review, see (13)]. Uysal et al. (14) introduced a match-like dual-task (DT) assessment for youth footballers and demonstrated that cognitive load significantly reduced agility performance while remaining unrelated to standard physical metrics, indicating that DT ability represents an independent performance domain. Similarly, Rezende and Praça (15) found that DT demands impaired tactical performance in small-sided games for all players, although more experienced athletes were less affected. In contrast, Praça et al. (16) reported that experience did not buffer the physical performance costs of DT (i.e., motor secondary tasks reduced physical output). Conversely, purely cognitive secondary tasks were not affected in experts–a discrepancy likely explained by differences in primary-task structure and high automatization even among less experienced players. Complementing these findings, Klotzbier and Schott (10) showed that under DT dribbling conditions, elite academy players were able to maintain their speed more effectively than amateur peers. In a study assessing speed performance under DT conditions with athletes of different sport types, Brinkbäumer et al. (9) found a significant performance decrement in a frequency-based tapping task across different sport types. Ice hockey and open-skill athletes lost performance to a smaller extent than closed-skill athletes. In general, the ability to perform under DT conditions might be an important performance indicator in open-sports (17) – more specifically, maintaining speed levels in perceptual-cognitive task environments is crucial for elite ice hockey athletes to maximize in-game performance.

DT training (DTT) might be a tool to enable athletes in this regard (11, 18–21). Athletes are purposefully exposed to DT scenarios, since chronic exposure seems to improve performance [for a review, see (13)]. Lucia et al. (11, 18–20) performed a series of studies with basketball players comparing five weeks of DTT to conventional training. Researchers employed similar training interventions across studies pairing concurrent motor (e.g., footwork, sprint, dribbling) and cognitive exercises (e.g., anticipation, discrimination, working-memory) aimed at improving functional abilities and cognitive functions. Cognitive stimuli were delivered with an LED system displaying symbols, letters, and numbers of different color, emitting sounds and interacting with athletes through proximity sensors. In semi-professional athletes, DTT led to improvements in sport-specific tests and larger gains in cognitive test accuracy (11), as well as faster dribbling speed, better accuracy, and faster response speed compared to a control group (18). Even in elite basketball players DTT led to improvements in sprint and agility, and reduced response times in a decision-making test, whereas the control group did not improve (19). Another study confirmed positive effects of DTT embedded into a physical training circuit. DTT group improved sprint completion time by 6% and agility by 4%, alongside increased motor anticipation (> 50%) and 10% faster response times in a cognitive discrimination task, whereas the conventional training group did not show these gains (20). Ramírez Lucas et al. (21) implemented an eight-week DTT program with U16 football players to test effects on physical (speed, agility), cognitive (working memory, planning), and technical (dribbling, passing) performance. The intervention entailed small-sided games or agility exercises with incorporated elements such as color identification, number sequences, mathematical operations, and auditory cues, which required the athletes to make quick decisions. The experimental group improved significantly across all assessed domains including DT cost, while the control group showed no change or declines. The authors conclude that brief, sport-specific DT training is an effective and time-efficient method to concurrently enhance physical, technical, and cognitive capabilities in youth football players. Summarizing, DTT seems to be a promising training tool. In order to improve game speed through DTT, the motor task should emphasize physical speed under concurrent cognitive demands. Increased efficiency in multitasking scenarios may originate in greater automatization of the speed task (22). However, a recent meta-analysis on the relationship between cognitive function, motor skills, and sports performance by Kalén and colleagues (23) proposes that traditional performance tests often do not reflect the complex requirements of real-game situations, rather, specificity in testing and training seems to be of great importance to ensure transfer to game performance. Typically, cognitive stimuli in most DT studies are non-specific (11, 18–21).

Virtual reality (VR) is increasingly used in sports training (24), to depict more sport-specific stimuli, and improve motor learning, visual perception, decision-making, and anticipation skills (25). VR training (VRT) targets both motor and cognitive skills, including balance, stability, sprinting, jumping, reaction time, perception-action coupling, as well as strategic, tactical, and decision-making skills (24, 26). VRT uniquely allows a high degree of specificity and standardization (27), making it a suitable tool for DT assessment and training (28). Ritter et al. (29) apply VRT in high-level athletes, by showing similar response types and time of elite karate athletes to attack moves either by a real or simulated. Heilmann and Schubert (30) examined whether sport-specific cognitive training in VR can improve inhibition in elite young ice hockey players. VRT was delivered using SenseArena© (Praha, Czech Republic) through drills that focused on cognitive functions, especially inhibition. Results showed significant improvements only in the sport-specific Flanker task for the VR-trained group, indicating enhanced inhibition in ecologically valid, hockey-related contexts, but no benefits in general cognitive tasks. Friedrich (32) compared a drill in an off-ice shooting rink and a similar drill in VR, also using SenseArena© (Praha, Czech Republic). VRT produced significantly higher levels of engagement and enjoyment compared to conventional drills, while some usability and perceived usefulness ratings lagged behind traditional methods. Bloechle et al. (32) developed a VR application to train professional ice hockey players to identify the goal's “largest exposed area” from the puck's perspective–a perceptual skill difficult to train with traditional methods. Real goalkeeper movements were captured via motion capture and replicated in VR. Thirty-four professionals participated: one group completed feedback-based VRT between two test sessions, while a control group trained without specific feedback. The feedback group showed significant post-training improvements in perceptual performance, and the usual performance decline (visual–puck misalignment) was eliminated in this group but not in controls. The authors conclude that short, targeted VRT with real-time feedback can effectively enhance sport-specific perceptual skills in high-performance settings. VRT seems to be a useful training tool, that allows exposure to DT scenarios with greater specificity of cognitive stimuli. However, no studies have yet examined the effects of DTT in VR on DT performance in ice hockey, nor compared its effects to regular DTT.

Physical warm-up is established as an acute performance enhancement in team sport (33). Accumulating evidence suggests that cognitive warm-up may also confer performance benefits (34–36). A “cognitive warm-up” preceding reading-training improved reading fluency and decoding in children, with gains mediated by enhancements in executive functions such as processing speed and inhibition (34). For example, a cognitive-based neuromuscular training improved dynamic balance and speed performance in youth soccer players, supporting that cognitive load integrated in training may enhance overall physical performance (35). This suggests that DTT or VRT may elicit positive effects on speed performance under DT conditions, however, evidence remains limited. Brinkbäumer et al. (9) highlighted substantial interindividual variability in athletes’ acute responses to DT load, ranging from performance decrements to slight improvements. Earlier work on trainability also suggests large interindividual variability in training adaptations (37–39). Since individual responsiveness is moderated through an interplay of endogenous and exogenous factors, alternative training regimes might overcome non-responsiveness (40). From an applied perspective, statistically significant changes do not necessarily imply practical relevance (41), and conversely non-significant changes may still be practically meaningful (42). What is most relevant for practitioners is whether training-induced changes are of a magnitude that exceeds the smallest effect considered practically worthwhile, commonly referred to as the smallest worthwhile change [SWC; (41, 43)]. This magnitude-based approach to interpreting training effects emphasizes the practical importance of observed changes rather than their statistical significance alone. Especially in applied settings that are constrained to small sample or trial sizes, SWC is an ideal analysis approach (44). It can be further supported by qualitative inferences about the observed effect sizes–namely, whether a change is likely to be certainly beneficial or detrimental, trivial, or unclear (45). Accordingly, the present study aimed (1) to test acute effects of VRT and DTT on DT speed performance in two complex cognitive-motor DT paradigms and (2) to explore potential moderating factors for positive or negative training adaptations on an individual basis. Therefore, in this study, we hypothesized that (1) VRT and DTT would produce positive effects on performance in at least some participants and (2) individual positive or negative responses would be moderated by metrics of physical, DT, and cognitive capacity.

## 2 Materials and methods

### 2.1 Subjects

The data was collected during a project funded by the German Federal Institute for Sport Science. Data was acquired during German national squad training camps or in home clubs of the players. Seventy-four German national squad ice hockey players (17.3 ± 1.7 years) participated in this study. Three different squads were included (U18 female: *n* *=* 19; U18 male: *n* *=* 32; U20 male: *n* *=* 23). All athletes confirmed mental and physical readiness and provided written informed consent before participating in the study. Healthy vision or correction (glasses or contact lenses) were required for participation.

### 2.2 Procedure

**Figure 1:**
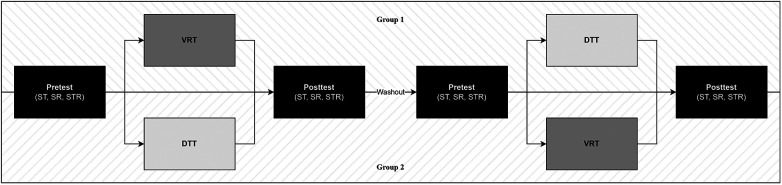
Cross-over study procedure. ST, simple tapping task; SR, speed-reading dual-task; STR, Stroop dual-task.

All participants performed one session of VRT and DTT in a randomized pre-post cross-over design. To investigate the acute effects of VRT and DTT on speed performance under DT conditions, all participants performed two DT paradigms with a focus on motor speed before and after the training sessions. Figure 1 depicts the study procedure all participants completed, either in Group 1 or Group 2.

### 2.3 Dual-task paradigms

The first paradigm followed Brinkbäumer et al. (9) and combined a simple tapping task (ST) with a secondary speed-reading task (SR). Tapping performance in the ST was recorded using a contact mat (Sport Voss. Doberschütz, Germany). Participants alternately tapped the mat with their left and right feet as quickly as possible for five seconds, initiated and terminated by acoustic signals. They began in a standardized stance, with both feet on the mat, knees slightly bent leaning forward, and arms held in front. Throughout the task, participants focused on a fixation cross displayed on a screen (Samsung SyncMaster 2494 HS; 100 cm front the mat, 133 cm high) to control visual attention across ST, SR and STR. This tapping task has been used to test athletes’ cyclical speed (46) and may be an easily implemented, sufficiently specific motor test option for team sports (9). In SR, simultaneous to the acoustic start signal, participants were presented with a honeycomb structure (63 totals combs in six rows with ten to eleven combs) on the screen in front of them. Twenty-eight combs were colored blue and the rest in red. In the center of all combs there was a number between one and nine. Participants were instructed to read all numbers in blue as fast as possible, from left to right, top to bottom. After all numbers were read measurement stopped. This paradigm aimed to replicate visual perception and verbal communication demands of ice hockey, since players must quickly recognize observed scenarios and verbalize them to teammates to improve collective perception (9). The second paradigm (STR) used the same tapping task but replaced the secondary cognitive component with a Stroop task (47). The Stroop task aims to assess selective attention and inhibitory control – two cognitive aspects that seems to be of relevance in dynamic team sports (31). After an auditory start signal, participants viewed a sequence of ten Stroop items on a screen and were instructed to name the color of the letters (not the word itself) as fast and accurate as possible. Each item appeared for 500 ms with a 50 ms interstimulus interval. The task ended once all items were processed. After a practice trial, two formal trials were completed, with a third if the results differed by > 10%. SR and STR stimuli were created and presented via PowerPoint (Microsoft, Washington, USA). The outcome variable of ST, SR and STR was the maximum tapping frequency (Hz), defined as the highest rate achieved within any one-second interval.

### 2.4 Dual-task training

In accordance with the performance demands of ice hockey, the motor tasks are speed-oriented, while the cognitive-perceptual tasks are primarily in the visual and vocal domains. The cognitive-perceptual stimuli are non-specific, similar to Formenti et al. (48), since they have been shown to be effective in improving cognitive skills, like reaction time, processing speed, and executive control. The DTT protocol combined hockey-specific motor actions with concurrent perceptual-cognitive challenges requiring rapid information processing and rule-based responding. All exercises consisted of continuous locomotion or puck-handling while simultaneously completing visually guided cognitive. In exercise 1, athletes controlled a puck around two cones arranged in a figure-eight pattern while performing peripheral visual jumps across designated points on a wall-mounted target board. Exercise 2 required athletes to read aloud sequences of colored circles presented on a “lying-eight” pattern and to skate around the cone matching the color of every second circle, thereby coupling continuous puck control with rapid visual decoding and response selection. In exercise 3, athletes executed puck-handling movements while performing serial saccades across alphabetic characters and skating around cones corresponding to the color associated with each read letter. Exercise 4 combined number reading with required motor responses: participants performed forehand or backhand rebound passes to the boards for even vs. odd numbers. This intervention primarily targets improvements in motor speed under DT conditions (see Supplementary Material for details). The training sessions were conducted in a sport-specific context using a stick and puck, but in standard sportswear, lasting approximately 20 min.

### 2.5 Virtual-reality training

VRT was performed with the VR system by SenseArena© (Praha Czech Republic), which presents immersive hockey-specific scenarios requiring rapid perceptual processing, decision-making, and motor execution (see Supplementary Material for details). The VR system is validated by professional players and coaches (31) and has been used in previous studies to provide a controlled, yet ecologically valid environment (30, 31). Athletes interact with a virtual environment designed to simulate ice hockey-specific perceptual challenges—such as visual search tasks and the identification of passing lanes or shooting opportunities—followed by decision-making tasks. They included a variety of game-like and task-based scenarios aimed at developing specific motor and cognitive skills. Training consisted of different exercises from task categories like “*Reaction*”, “*Reading*”, “*Multitasking*”, “*Player Tracking*” (e.g., *“Find the Line”, “Multiple Object Tracking”, “Shot Lane Recognition”*) that integrated stickhandling and passing with complex visual stimuli. In “Find the Line”, athletes identified a dynamically moving pass window formed by two virtual goals and delivered timed passes to a moving teammate; difficulty was manipulated through pass-window width, movement speed, positional constraints, and time limits, with athletes typically increasing both attempt volume and efficiency over time. In “Multiple Object Tracking”, athletes tracked two to four colored teammates among distractors while simultaneously controlling the puck and matching the pass target to the previously displayed color; task difficulty varied according to the number of tracked objects, which directly influenced efficiency and error rates across training progression. In “Shot Lane Recognition,” participants identified and passed into the correct shooting lane based on color cues from moving players while managing puck control, with difficulty increased via pass-speed adjustments that reduced available decision time; performance typically improved with higher pass frequencies and accuracy across sessions. All VRT tasks provided immediate visual and auditory feedback and allowed fine-grained manipulation of complexity, enabling systematic overload of perceptual-cognitive resources in a controlled yet ecologically valid environment. This intervention is primarily designed to enhance perceptual processing by improving both the speed and capacity of information processing. VRT sessions were likewise conducted in standard sportswear and lasted approximately 20 min.

### 2.6 Related performance outcome measurements

Besides DTT and VRT interventions, athletes conducted an extensive performance diagnostic. If athletes displayed distinct responses to either intervention, the relationship between intervention effects and performance tests (cognitive performance, simple on-ice speed, complex on-ice speed) were analyzed, to further explore how both are related.

For cognitive performance two-choice reaction time (2CRT), stop-signal reaction time (SSRT), processing speed, sustained attention, and spatial working memory was measured as described by Brinkbäumer et al. (9). For each test, the percentile rank within all participating athletes was calculated for each athlete individually. Individual test percentile ranks were averaged for all cognitive tests to form a composite cognitive performance score. Spatial working memory (Corsi) was factored in once, as the average between forward and backward Corsi span. Processing speed was measured with the Zahlen-Verbindungs-Test [ZVT; (49)], a paper-and-pencil task in which participants connect numbers from 1 to 90 as quickly and accurately as possible within 30 s. The test comprised four sheets, and performance was calculated as the mean number of correctly connected numbers across sheets (50). Sustained attention was assessed using the d2-R test (d2-R) (51), which measures concentration under time pressure. Participants marked target stimuli (the letter “d” with two dashes) among distractors across 14 lines, each completed within 20 s. The dependent variable was sustained attention performance, derived from correct responses minus errors, with higher values indicating better performance. Spatial working memory was evaluated using the forward and backward Corsi Block Test (52), administered digitally via PsyToolkit (53, 54) on a laptop. Participants reproduced sequences of illuminated blocks in either the same or reverse order. Sequence length increased with correct responses and ended after two consecutive errors or successful completion of all nine blocks. The mean number of correctly recalled items served as the outcome measure. Response inhibition was assessed using a stop-signal task following Verbruggen and Logan (55), with modifications by Heppe and Zentgraf (56). Participants responded to left- or right-pointing arrows in go trials and attempted to inhibit their response in stop trials, which occurred in 25% of cases when the arrow changed color after a variable stop-signal delay. An adaptive staircase procedure adjusted stop-signal delay to maintain approximately 50% inhibition success. SSRT was calculated using the integration method, and 2CRT was recorded in the process of determining SSRT and included as an additional outcome. Testing was conducted individually under supervision.

On-ice speed was assessed in four test paradigms entailing a simple (onST) and complex test (CT):

1. In the onST ’Sprint’, athletes complete a 30-m linear on-ice sprint, timed using photocells (Microgate®, Bolzano, Italy). The linear sprint test is a typical diagnostic tool for acceleration and speed in ice hockey (57). The test setup was similar to the 30-m sprint in the “ice hockey specific complex test” described by Bloch et al. (58). Players initiate the sprint from a split stance positioned one meter before the first photocell, with the lead skate placed just behind the start line. In the CT ’Sprint-L’, seven LED lights are positioned along the sprint track and illuminate sequentially (start to finish) in randomized colors (red, blue, yellow). Immediately after completing the sprint, participants verbally reproduce the color sequence to verify task compliance. Equal emphasis is placed on skating and cognitive performance.
2. The ‘Change of Direction Test’ (CODT) is an ice-hockey-specific adaptation of the team-sport protocol described by Willberg et al. (59). In the onST condition, participants navigate a predefined change of direction course involving multiple angles (see Supplementary Material for illustration). Starting one meter before the photocell in a split stance, players maintain forward gaze while approaching four cones in alternating forward and backward skating, fully passing each cone while holding their stick. The CT condition (CODT-V) incorporates a visual-verbal working memory task. During the course, athletes view a screen located five meters behind the rear cones displaying a sequence of eight digits (“1–8”), which they must verbally recall immediately upon trial completion. As in the other tests, cognitive and skating components are given equal priority.
3. In the ‘Lateral Shuttle’ (LS), players perform continuous lateral skating and side-cutting maneuvers between two parallel one-meter lines spaced five meters apart as onST, which is a common ice-hockey diagnostic (e.g., (60); see Supplementary Material for illustration). Beginning with the right skate on one line, athletes complete as many shuttles as possible within 15 s, ensuring contact with each line on every turn. All trials are filmed at 30 Hz and analyzed using Filmora X (Wondershare Technology Co., Shenzhen, China). The CT condition (LS-P) incorporates puck handling and four LED-illuminated goal targets. While laterally shuttling and dribbling, players monitor a visual cue that activates one of the four goals after seven to ten seconds, prompting an immediate shot. Accurate detection and response are assessed, and mean shuttle time prior to cue onset is calculated.
4. The ‘Transition Agility Test’ (TAT), conducted both with (TAT-P; CT) and without a puck (onST), follows the protocol described by Bloch et al. (58). Starting one meter before the photocell, players accelerate forward, transition into backward skating via a pivot, and alternate directional changes between pylons until crossing the finish line. Completion time is recorded using photocells (Microgate®, Bolzano, Italy).

Analogous to the procedure for the composite cognitive performance, an individual percentile rank was calculated for each onST and CT test. These percentile ranks were then averaged to form two composite scores: one for onST and one for CT performance.

### 2.7 Statistical analysis

Data is reported in *M* ± *SD*. For statistical analysis SPSS Statistics Version 26 (IBM Corporation, Armonk, USA) was used. The level of significance was set at *p* < .05. Normal distribution of the data was tested through Kolmogorov–Smirnov test and visual inspection of the Q-Q plot. Outliers were assessed through studentized residuals. A two-way repeated measures ANOVA was conducted to examine the effect of intervention (DTT vs. VRT) and time (pre, post) on tapping ST, SR, and STR performance. To quantify the individual response of the athletes, individual SWC was calculated according to Turner et al. (61) using Excel (Microsoft, Redmond, USA):

$$Certain\;change=\;2*\frac{\emptyset \;all\;trials\;across\;one\;test}{100}*\;\emptyset \;Coefficient\;of\;variance\;$$

Participants were clustered into three categories: 1) benefit, 2) no change, and 3) decrement. A correlation analysis between pre-post differences for ST, SR and STR was calculated for both training interventions and related performance outcome measures was calculated to further examine their relationship.

## 3 Results

**Figure 2:**
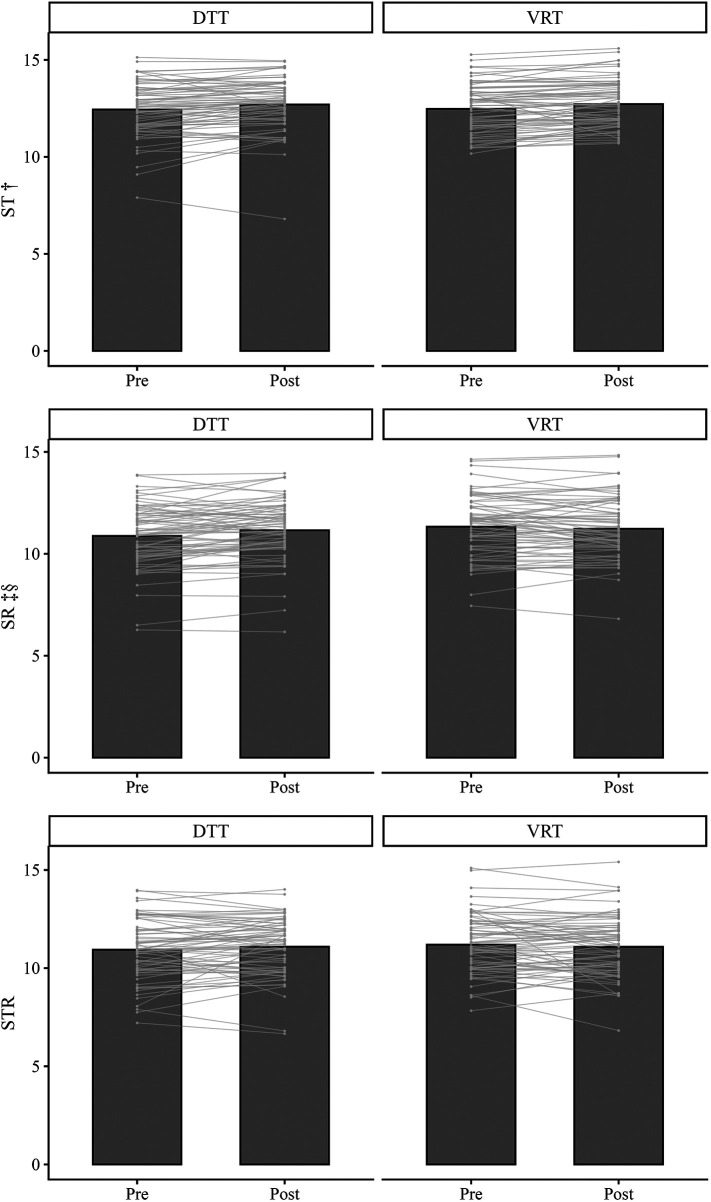
Intervention effects of DTT and VRT in ST, SR, and STR. DTT, dual-task training; VRT, virtual-reality training; ST, simple tapping task; SR, speed-reading dual-task; STR, Stroop dual-task. Bar graph=mean value, line graphs=individual values, † = significant main effect of time, ‡ = significant main effect of intervention, § = significant interaction effect.

In the DTT condition, performance in ST, SR, and STR increased from pre to post, whereas in the VRT condition ST increased, but SR and STR performance decreased (Table 1). Individual performance trajectories are depicted in Figure 2.

**Table 1:** Tapping performance across paradigms and interventions.

Paradigm	Pre [Hz]	Post [Hz]	Difference [Hz]
DTT ST	12.44 ± 1.34	12.7 ± 1.30	0.27 ± 0.61
VRT ST	12.47 ± 1.24	12.72 ± 1.18	0.24 ± 0.57
DTT SR	10.87 ± 1.47	11.16 ± 1.42	0.28 ± 0.70
VRT SR	11.33 ± 1.45	11.23 ± 1.44	−0.10 ± 0.68
DTT STR	10.94 ± 1.51	11.09 ± 1.42	0.15 ± 0.88
VRT STR	11.19 ± 1.46	11.08 ± 1.49	−0.11 ± 0.94

DTT, dual-task training; VRT, virtual-reality training; ST, simple tapping task; SR, speed-reading dual-task; STR, Stroop dual-task.

A two-way repeated measures ANOVA was conducted to examine the effect of intervention (DTT vs. VRT) and time (pre, post) on ST, SR, and STR performance. Kolmogorov–Smirnov test (*p* > .05) and visual inspection of Q-Q plot confirmed normal distribution of the data. There was one outlier, as indicated by standardized residuals greater than ±3. It was inspected and identified as a genuine outlier. Analysis was run without the outlier and since it did not influence the results it was kept in the data. For ST, there was a significant main effect of time, *F*(1, 73) = 20.731, *p* < .001, *η²_p_* = .221, but no significant main effect of intervention on performance, *F*(1, 73) = .900, *p* = .765, *η²_p_* = .001. Additionally, the interaction between intervention and time was not significant, *F*(1, 73) = .033, *p* = .857, *η²_p_* = .000. For SR, there was no significant main effect of time, *F*(1, 73) = 2.039, *p* = .158, *η²_p_* = .027, but a significant main effect of intervention on performance, *F*(1, 73) = 5.647, *p* = .020, *η²_p_* = .072. Additionally, the interaction between intervention and time was significant, *F*(1, 73) = 15.273, *p* < .001, *η²_p_* = .173, indicating slight decrement through VRT and improvement through DTT. For STR, there were no significant main effects of time, *F*(1, 73) = .071, *p* = .790, *η²_p_* = .001 and intervention on performance, *F*(1, 73) = 1.320, *p* = .254, *η²_p_* = .018 and the interaction between intervention and time was not significant, *F*(1, 73) = 3.653, *p* = .470, *η²_p_* = .048.

Individual changes according to SWC (2*CV) are depicted in Table 2. The majority of individuals did not show certain change through either intervention. Through DTT more individuals experienced certain benefit than decrement for ST, SR, and STR. Through VRT there were more certain benefits than decrements for ST only. In SR and STR cases of certain performance decrements outnumbered benefits.

**Table 2:** Clusters of individual changes based on SWC.

Paradigm	Benefit	No change	Decrement
DTT ST	*n* = 13	*n* = 58	*n* = 3
VRT ST	*n* = 9	*n* = 63	*n* = 2
DTT SR	*n* = 12	*n* = 61	*n* = 1
VRT SR	*n* = 6	*n* = 61	*n* = 7
DTT STR	*n* = 9	*n* = 61	*n* = 4
VRT STR	*n* = 5	*n* = 61	*n* = 8

DTT, dual-task training; VRT, virtual-reality training; ST, simple tapping task; SR, speed-reading dual-task; STR, Stroop dual-task.

For the related performance outcome measurements, the time for Sprint was 4.42 ± 0.29 s and 4.68 ± 0.35 s for Sprint-L. The CODT averaged 9.28 ± 0.68 s and the CODT-V 9.58 ± 0.71 s. The TAT yielded a mean of 13.90 ± 0.81 s and the TAT-P 14.77 ± 1.08 s. LS averaged 1.63 ± 0.22 s and LS-P 1.66 ± 0.22 s. 2CRT was 455.93 ± 90.19 ms and SSRT 230.97 ± 46.00 ms. The ZVT had a mean of 47.94 ± 7.43 and d2-R averaged 173.60 ± 27.96. Mean Corsi fwd. was 5.93 ± 1.01 and Corsi bwd. was 5.59 ± 1.04. Correlation between pre-post differences for ST, SR, and STR for DTT and VRT are shown in Table 3. However, some athletes who completely performed the intervention were not able to perform all test of related performance outcome measurements, because of diverse reasons (e.g., leaving the training camp early, load management, injuries). Since Kolmogorov–Smirnov test revealed a violation of normal distribution for several variables (*p* < .05), Spearman's rho was calculated.

**Table 3:** Spearman's correlation between intervention effects and related performance outcome measurements.

Test		DTT difference			VRT difference		
	*n*	ST	SR	STR	ST	SR	STR
Sprint	63	0.024	−0.001	0.106	0.014	0.017	0.072
Sprint-L	63	−0.059	−0.178	0.16	−0.094	−0.013	0.136
CODT	63	0.033	0.036	.270*	0.026	−0.082	0.09
CODT-V	62	0.008	−0.093	.392**	0.066	−0.014	0.118
TAT	63	−0.012	0.081	.271*	−0.007	0.004	0.169
TAT-P	63	0.064	0.023	.341**	0.036	0.042	0.159
LS	62	0.163	0.057	.339**	0.038	0.084	0.13
LS-P	62	0.063	−0.083	0.198	0.064	0.138	0.198
2CRT	63	−0.05	−0.192	−0.08	0.128	0.041	0.014
SSRT	63	−0.058	0.037	−0.019	−0.186	−0.158	−0.053
ZVT	62	-.250*	−0.128	−0.049	−0.174	−0.118	−0.113
d2-R	63	−0.176	0.081	0.133	**-**.353**	0.038	−0.004
Corsi fwd.	60	**.** 299*	0.183	0.119	−0.138	0.016	0.137
Corsi bwd.	60	0.171	0.061	0.103	−0.098	0.06	0.112

DTT, dual-task training; VRT, virtual-reality training; ST, simple tapping task; SR, speed-reading dual-task; STR, Stroop dual-task; Sprint-L,  Sprint with Lights; CODT, Change of Direction Test; CODT-V, Change of Direction Test with Visuo-Verbal Task; LS, Lateral Shuttle; LS-P, Lateral Shuttle with Puck; TAT, Transition Agility Test; TAT-P, Transition Agility Test with Puck; 2CRT, two-choice reaction time; SSRT, stop-signal reaction time; ZVT, processing speed; d2-R, sustained attention. Bold indicates statistical significance. ** < .01, * < .05.

Relationship between intervention effects and related performance outcome measurements for most prominent responders is displayed in Table 4. Besides the included 14 athletes there were:

- Twenty-nine (10 females, 19 males) athletes with no change in ST, SR, and STR across both interventions,
- Nine (2 females, 7 males) athletes with certain benefit in one of the paradigms through DTT
- Nine (3 females, 6 males) athletes with certain benefit in one of the paradigms through VRT
- Six (2 females, 4 males) athletes with certain benefit in one of the paradigms through DTT and VRT
- Four (2 females, 2 males) athletes with certain decrement in one of the paradigms through VRT
- One male athlete with certain decrement in one of the paradigms through DTT
- Two male athletes with certain decrement in one of the paradigms through DTT and VRT.

**Table 4:** Relationship between prominent responders and related performance outcome measurements.

		DTT			VRT			Performance scores		
Response	Athlete	ST	SR	STR	ST	SR	STR	Cognitive	onST	CT
1	1	+	+	=	+	+	+	17%	65%	43%
2	2	+	+	+	=	=	=	64%	60%	41%
	3	+	=	+	=	=	=	n.d.	n.d.	n.d.
	4	+	+	=	=	=	=	29%	34%	39%
	5	=	+	+	=	=	=	58%	37%	39%
3	6	=	=	=	+	=	+	n.d.	n.d.	n.d.
4	7	+	+	=	=	=	-	55%	39%	56%
	8	+	+	=	=	-	-	37%	51%	65%
5	9	+	=	-	=	=	=	52%	30%	17%
6	10	=	-	+	=	-	=	46%	21%	10%
	11	-	=	+	=	-	=	n.d.	n.d.	n.d.
	12	+	=	-	=	-	-	53%	62%	48%
7	13	=	=	=	=	-	-	62%	71%	84%
8	14	=	-	-	-	-	=	44%	92%	50%

DTT, dual-task training; VRT, virtual-reality training; ST, simple tapping task; SR, speed-reading dual-task; STR, Stroop dual-task; onST, on-ice simple speed task; CT, on-ice complex speed task. 1=benefit through DTT and VRT, 2=benefit through DTT only, 3=benefit through VRT only, 4=benefit through DTT and decrement through VRT, 5=mixed results through DTT, 6=mixed results through DTT and decrement through VRT, 7=decrement through VRT, 8=decrement through DTT and VRT, “+”=certain benefit, “-”=certain decrement, “=”=no certain change, n.d.=no data.

Clear VR benefits were present in athletes 1 and 6, although only athlete 1 had complete accompanying data. Athlete 1 (male, 19.2 years) showed DTT benefits in SR and ST, with no change in STR. In VR, this athlete demonstrated benefits in all three paradigms. Cognitively, this athlete had comparatively low scores (2CRT 30%, SSRT 25%, ZVT 26%, d2-R 0%, Corsi 5%). onST were moderate to good (Sprint 84%, CODT 59%, TAT 77%, LS 40%). CT performance was moderate (Sprint-L 39%, CODT-V 46%, TAT-P 43%, LS-P 44%).

Clear DTT benefits were present in athletes 2, 3, 4, 5, although there is no data on related performance outcome measurements for athlete 3. Athlete 2 (male, 19.3 years) exhibited consistent DTT benefits across all DT paradigms, while VR performance showed no change. Cognitive test scores were moderate to high (ZVT 85%, d2-R 43%, Corsi 43%), but 2CRT and SSRT are missing. onST performance was mixed with good Sprint (94%) and LS (72%), but lower CODT (36%) and TAT performance (39%). CT performance was moderate (Sprint-L 32%, CODT-V 30%, TAT-P 64%, LS-P 40%). Athlete 4 (male, 18.9 years) showed DTT benefits for SR and ST with no change in STR. All VR paradigms showed no change. Cognitively, this athlete scored low to moderate (2CRT 2%, SSRT 47%, ZVT 41%, d2-R 39%, Corsi 18%). onST performances were low to moderate (CODT 2%, TAT 30%, LS 14%). Only Sprint was above average (90%). CT outcomes were in the lower range (CODT-V 25%, TAT-P 32%, LS-P 12%), except from Sprint-L (89%). Athlete 5 (male, 17.0 years) demonstrated benefits through DTT in SR and STR and no change in ST; all VR paradigms showed no change. Cognitive scores were moderate to high (2CRT 75%, ZVT 54%, d2-R 79%, Corsi 50%), except for SSRT (32%). onST performance was low to moderate (Sprint 34%, CODT 48%, TAT 32%, LS 33%). CT outcomes were also low to moderate (Sprint-L 36%, CODT-V 41%, TAT-P 23%, LS-P 56%).

Athletes 7 and 8 displayed mixed effects through DTT, showing improvement in one paradigm but decrements or no changes in another. VR changes in these athletes were neutral or decremental. Athlete 7 (male, 19.3 years) experienced benefits through DTT in SR and ST with no change in STR. In contrast, VR performance was characterized by a decrement in STR, while SR and ST remained unchanged. Cognitively, he scored moderate to high (2CRT 80%, ZVT 69%, d2-R 66%, Corsi 50%), only SSRT was below average (11%). onST outcomes were moderate (Sprint 48%, CODT 39%, TAT 25%, LS 44%). CT performance was comparatively better (Sprint-L 68%, CODT-V 50%, LS-P 88%), apart from TAT-P (16%). Athlete 8 (male, 20.3 years) showed improvements two paradigms through DTT (ST, SR) and VRT (SR, STR) with no changes in the remaining paradigm. Cognitive performance was in the moderate range (ZVT 34%, d2-R 44%, Corsi 34%), but 2CRT and SSRT are missing. onST and CT performance was heterogenous, with good results for CODT (77%), LS (70%), CODT-V (91%) and LS-P (77%), moderate for Sprint (37%) and Sprint-L (59%), but low for TAT (18%) and TAT-P (34%).

Athlete 9 (male, 18.9 years) displayed mixed results through DTT, with no change in SR, a decrement in STR, and a benefit in ST. VRT showed no change. Cognitive performance was relatively high (2CRT 89%, SSRT 20%, ZVT 87%, d2-R 50%, although Corsi was lower at 14%). onST results were low to moderate (Sprint 52%, CODT 41%, TAT 0%, LS 26%). CT performance was below average (Sprint-L 9%, CODT-V 34%, TAT-P 2%, LS-P 21%).

Athletes 10, 11, 12 showed mixed effects through DTT and decrements in one or two paradigms through VRT. Athlete 10 (male, 16.8 years) showed decrement in SR, benefit in STR, and no change in ST after DTT. With VRT, he showed a decrement in SR, while STR and ST remained unchanged. Average cognitive performance moderate, but individual results were heterogenous with good results for (2CRT 97%, ZVT 61%, d2-R 55%, Corsi 14%) and low performance in SSRT (4%). onST and CT outcomes were generally low (Sprint 37%, CODT 7%, TAT 18%, LS 23%, Sprint-L 0%, CODT-V 2%, TAT-P 5%, LS-P 33%). There is no data on related performance outcome measurements for athlete 11. Athlete 12 (male, 19.3 years) showed mixed results through DTT. There was no change in SR, decrement in STR, and benefit in ST. VRT resulted in decrements in SR and STR with no change in ST. Cognitive performance was moderate (2CRT 39%, SSRT 50%, ZVT 79%, d2-R 73%, Corsi 25%). onST output was comparatively good (Sprint 60%, CODT 25%, TAT 61%, LS 100%). Average CT results were moderate but heterogenous, with tests without puck below and test with puck above average (Sprint-L 7%, CODT-V 7%, TAT-P 77%, LS-P 100%).

Athlete 13 (male, 19.0 years) showed no change in through DTT across all three paradigms, but VRT lead to decrements in SR and STR, with ST unchanged. Cognitive performance was moderate to high (2CRT 48%, SSRT 52%, ZVT 90%, d2-R 39%, Corsi 81%), resulting in the highest composite score. onST outcomes were strong (Sprint 90%, CODT 50%, TAT 91%, LS 54%). CT performance was excellent (Sprint-L 100%, CODT-V 57%, TAT-P 89%, LS-P 91%).

Athlete 14 (male, 20.3 years) exhibited decrements through DTT and VRT. With DTT there was a decrement in STR with no change in SR and ST. With SR and ST showed decrements, while STR remained unchanged. Cognitive scores were heterogenous ranging from low (d2-R 3%, Corsi 25%) to moderate (2CRT 77%, SSRT 48%, ZVT 67%). onST performance was high (Sprint 95%, CODT 84%, TAT 96%, LS 93%). Average CT performance was moderate but heterogenous with low performance in Sprint-L (18%) and LS-P (28%), moderate performance in TAT-P (68%) and high performance in CODT-V (84%).

## 4 Discussion

The present study aimed to examine the acute effects of DTT and VRT on speed performance under cognitively demanding conditions in elite youth ice hockey players and explore individual differences in training response as a function of physical and cognitive performance capacities. Using two cognitively demanding DT paradigms, the study also aimed to explore individual differences in training response, considering both physical and cognitive performance capacities. While both interventions led to small improvements in ST, only DTT showed a positive effect on performance in SR. In contrast, neither intervention improved performance in the STR, with VRT even resulting in performance decrements in some individuals.

Both DTT and VRT led to statistically significant, albeit small, improvements in the ST tapping paradigm. This suggests that even short, 20-minute training sessions can acutely enhance basic motor execution in elite athletes. While the task itself lacked a cognitive component, the physical engagement during both interventions may have increased neuromuscular readiness or arousal, similar to known benefits of physical warm-up routines (33). From a practical perspective, this finding indicates the potential of incorporating DTT or VRT into pre-competition warm-ups to prime basic motor speed. However, given that no differential effects between interventions were found, cognitive engagement or task complexity may play a more decisive role in determining training effectiveness (22).

In SR, DTT resulted in a statistically significant improvement, while VRT led to a small decline in performance. This pattern suggests that DTT may be more effective than VRT in helping athletes maintain motor performance under moderate cognitive load. The differing effects of DTT and VRT might stem from how closely each intervention matches the cognitive demands of SR. DTT combines visual, verbal, and motor elements concurrently, reinforcing the integration of cognitive processing with movement execution, which might facilitate improved cognitive-motor coordination (22). This congruence with the SR paradigm might enhance attentional resource allocation and sensorimotor coordination. In contrast, while VRT emphasizes perceptual-cognitive skills in realistic simulations (24, 25), its effects may not transfer well to performance in tasks with a high degree of verbal, linguistic, or rule-based cognitive demands such as the SR paradigm. The slight decline after VRT might suggests a mismatch between dominant cognitive processes trained and those required in SR. This reinforces the importance of task-specificity in training – a principle emphasized by Kalén et al. (23) – suggesting that cognitive training needs to closely mirror the performance context to yield positive transfer. Practically, DTT may be more suited for acute preparation in scenarios where cognitive and motor challenges coexist such as high-tempo game situations in ice hockey.

Neither DTT nor VRT produced significant improvements in STR, which placed greater demands on executive functions such as inhibition and cognitive flexibility (47). Notably, more athletes experienced performance decrements following VRT than DTT in this paradigm. These findings suggest that a single training session may be insufficient to enhance performance under high cognitive load – a limitation consistent with prior research showing performance decline in elite athletes under DT conditions (9). The fact that VRT was particularly associated with more decrements may reflect cognitive interference or fatigue due to mismatch between the cognitive tasks trained (i.e., perception-action, decision-making) and those tested (i.e., verbal inhibition, rapid switching). This finding aligns with the need for better alignment between training stimuli and test paradigms (23). Moreover, the results underscore the importance of individual response profiling in high-performance settings, where uniform training approaches may not benefit all athletes equally (61).

Beyond these group-level findings, the SWC-based classification highlights marked interindividual variability in acute training responses. Across all paradigms and both interventions, the majority of athletes fell into the “no certain change” category, with only a minority showing clear benefits or decrements. This pattern aligns with earlier work on heterogeneous trainability in physiological traits (38, 39) and supports the view that even under standardized conditions, athletes differ substantially in how they acutely respond to a given stimulus (40, 61). Practically, this means that neither DTT nor VRT can be assumed to be uniformly advantageous as a pre-game warm-up: while some players may experience meaningful acute performance gains, others show trivial or even negative responses, particularly under higher cognitive load. An underlying factor of these heterogenous responses and a limitation to the group-level findings, might be differing internal physical and cognitive load across the participants during the interventions. Number of exercises and intervention duration was identical for VRT and DTT to match external load between interventions, but the individual response was not assessed.

The correlation analysis offers some first, albeit exploratory, insights into the influence of related performance outcome measurements on individual responses. For DTT, improvements in STR were positively associated with several on-ice speed measures (CODT, CODT-V, TAT, TAT-P, LS), although these associations were small to moderate in magnitude and should be interpreted as exploratory. This might suggest that athletes who are already proficient in game-like skating tasks – especially those requiring rapid direction changes, transitions, and puck handling – may be better positioned to acutely benefit from DTT when facing executive-function demands, such as those embedded in the Stroop paradigm. One could speculate that higher on-ice speed might reflect a more automatized motor repertoire and/or more efficient cognitive-motor integration, which could free up resources to deal with additional cognitive load (22). The positive association between STR improvements and spatial working memory (Corsi fwd.), together with the negative correlation with processing speed (ZVT), may indicate that athletes with stronger visuospatial updating but not necessarily superior generic speed of processing gain more from DTT in highly demanding DT situations. However, the effect sizes of these correlations are mostly small to moderate, and no corrections for multiple comparisons were applied, so these patterns must be interpreted cautiously and treated as hypothesis-generating rather than confirmatory.

For VRT, the correlational pattern was even less systematic. The only significant association indicated that athletes with lower sustained attention (d2-R) tended to show larger gains in ST after VRT, while no robust relationships with complex speed or other cognitive measures emerged. One speculative interpretation is that VRT, with its immersive and highly engaging stimulus environment (25, 31), may acutely increase arousal and attentional focus in athletes who initially struggle with sustained attention, thereby facilitating simple motor output. Conversely, athletes with already high attentional capacities might experience VRT as an additional cognitive load that does not translate into immediate benefits in a non-specific tapping task and may even induce short-term interference or fatigue. Taken together, the correlation matrix supports the notion that DTT effects in complex DT conditions are more tightly linked to existing complex motor and visuospatial abilities, whereas VRT effects in this acute context are less clearly moderated by the tested variables.

The individual case analyses further illustrate the complexity and non-linearity of these relationships. For example, athlete 1 who benefited from both DTT and VRT across most paradigms had comparatively low cognitive test scores but moderate to good in onST and moderate in CT, suggesting that athletes with “headroom” in cognitive capacity and solid but not exceptional skating proficiency may profit from both types of interventions. In contrast, athlete 13, who showed decrements after VRT despite strong cognitive and on-ice profiles, exemplifies a high-performing athlete for whom an additional, non-specific cognitive-perceptual load shortly before testing may be detrimental, possibly due to cognitive saturation or disrupted pre-existing routines. DTT-only responders (e.g., athletes 2, 4, 5) tended to present with moderate-to-good cognitive profiles and heterogeneous onST and CT performance, which may favor a training mode that tightly couples sport-specific motor demands with relatively simple visual–verbal stimuli. Conversely, athletes displaying mixed or negative responses, particularly to VRT (e.g., athletes 10, 12, 14), often combined uneven cognitive profiles with lower on-ice performance, suggesting that for some athletes, a single VR session may impose excessive cognitive-motor demands relative to their current capacity.

Importantly, these individual patterns do not support a simple “one-size-fits-all” responder profile. Instead, they point toward a nuanced interaction between cognitive capacity, simple, and complex on-ice speed, and the specific characteristics of the intervention. From an applied perspective, this argues for a more individualized selection of cognitive-motor warm-up formats in elite ice hockey. Athletes with strong complex skating skills and good visuospatial working memory may be promising candidates for DTT-based warm-ups when the goal is to preserve performance under substantial cognitive load, whereas VRT might be more useful acutely for athletes who show deficits in attentional engagement but are not immediately exposed to high-level linguistic or inhibitory demands. At the same time, the present data come from a single-session design and relatively small subgroups in the case analysis, so any such recommendations should be considered preliminary. Future studies with larger samples, longitudinal interventions, and more fine-grained profiling are needed to confirm whether stable “responder phenotypes” to DTT and VRT exist and to determine how these can be leveraged to individualize cognitive-motor warm-up strategies in elite team sports.

## 5 Conclusion

This study demonstrates that acute DTT is more effective than VRT in supporting tapping speed performance under cognitive load in elite youth ice hockey players. While both interventions improved tapping without additional cognitive load, only DTT enhanced tapping performance when cognitive and motor demands were combined. VRT, despite its ecological and perceptual advantages, did not translate to improved DT tapping performance. The pronounced heterogeneity in individual responses underscores that cognitive-motor warm-up strategies cannot be uniformly applied across athletes. Instead, practitioners should consider individualized profiles – including complex on-ice speed capacities and cognitive characteristics – when integrating DTT or VRT into pre-performance routines. Future studies should examine longitudinal adaptations, refine task-specific cognitive stimuli, and explore whether personalized cognitive-motor interventions can optimize game speed performance in elite ice hockey.

## Data Availability

The data analyzed for the purposes of this study was part of a multidisciplinary large-scale data set, which included multiple points of measurement and a cross-sectional and interventional perspective. The subset of data included in this study covered the cross-sectional data collected in the period 2021–2022. The raw data supporting the conclusions of this article will be made available by the authors, without undue reservation.

## References

[1] Schulze S Laudner KG Delank KS Brill R Schwesig R. Reference data by player position for an ice hockey-specific complex test. Appl Sci. (2021) 11(1):280. 10.3390/app11010280

[2] Bond CW Bennett TW Noonan BC. Evaluation of skating top speed, acceleration, and multiple repeated sprint speed ice hockey performance tests. J Strength Cond Res. (2018) 32(8):2273–83. 10.1519/JSC.0000000000002644 29878985

[3] Silvestri MA Cleather DJ Callaghan S Perri J Legg HS. Examining the determinants of skating speed in ice hockey athletes: a systematic review. J Strength Cond Res. (2025) 39(4):507–14. 10.1519/JSC.0000000000005054 40153565

[4] Vigh-Larsen JF Beck JH Daasbjerg A Knudsen CB Kvorning T Overgaard K Fitness characteristics of elite and subelite male ice hockey players: a cross-sectional study. J Strength Cond Res. (2019) 33(9):2352–60. 10.1519/JSC.0000000000003285 31343551

[5] Hughes W Healy R Lyons M Nevill A Higginbotham C Lane A The effect of different strength training modalities on sprint performance in female team-sport athletes: a systematic review and meta-analysis. Sports Med. (2023) 53(5):993–1015. 10.1007/s40279-023-01820-5 36877405

[6] Edouard P Mendiguchia J Guex K Lahti J Prince C Samozino P Sprinting: a key piece of the hamstring injury risk management puzzle. Brit J Sport Med. (2023) 57(1):4–6. 10.1136/bjsports-2022-105532 35927000

[7] Murphy A Burgess K Hall AJ Aspe RR Swinton PA. The effects of strength and conditioning interventions on sprinting performance in team sport athletes: a systematic review and meta-analysis. J Strength Cond Res. (2023) 37(8):1692–702. 10.1519/JSC.0000000000004440 37494121

[8] Sheppard JM Young WB. Agility literature review. J Sport Sci. (2006) 24(9):919–32. 10.1080/02640410500457109 16882626

[9] Brinkbäumer M Kupper C Reichert L Zentgraf K. Dual-task costs in speed tasks: a comparison between elite ice hockey, open-skill and closed-skill sports athletes. Front Psychol. (2024) 10:1357312. 10.3389/fpsyg.2024.1357312 PMC11284104 39077212

[10] Klotzbier TJ Schott N. Skillful and strategic navigation in soccer – A motor-cognitive dual-task approach for the evaluation of a dribbling task under different cognitive load conditions. Front Psychol. (2024) 15:1356892. 10.3389/fpsyg.2024.1356892 38933580 PMC11205518

[11] Lucia S Bianco V Boccacci L Di Russo F. Effects of a cognitive-motor training on anticipatory brain functions and sport performance in semi-elite basketball players. Brain Sci. (2022) 12(1):102302. 10.3390/brainsci12010068 PMC8773627 35053809

[12] Koch I Poljac E Müller H Kiesel A. Cognitive structure, flexibility, and plasticity in human multitasking – an integrative review of dual-task and task-switching research. Psychol Bull. (2018) 144:557–83. 10.1037/bul0000144 29517261

[13] Moreira PED Dieguez GT de Oliveira B da Glória Teles S Praça GM. The acute and chronic effects of dual-task on the motor and cognitive performances in athletes: a systematic review. Int J Env Res Public Health. (2021) 18:1732. 10.3390/ijerph18041732 33579018 PMC7916747

[14] Uysal Ö Atalay Güzel N Bayrakcı Tunay V Fırat T. A novel method to measure dual-task capacity in young football players: a preliminary study. J Athl Training. (2024) 59(12):1197–202. 10.4085/1062-6050-0210.24 PMC11684746 39180151

[15] Rezende VHS Praça GM. Adopting dual-tasks in small-sided games training in youth soccer: the influence of experience level on tactical performance. Int J Sports Sci Coach. (2024) 20(1):139–51. 10.1177/17479541291372

[16] Praça GM de Almeida Oliveira PH Santos Resende VH. Dual-tasks in soccer: effects of players’ experience and task condition on physical performance. Percept Motor Skill. (2024) 131(4):1378–97. 10.1177/00315125241257398 38804982

[17] Schaefer S Scornaienchi D. Table tennis experts outperform novices in a demanding cognitive-motor dual-task situation. J Motor Behav. (2020) 52(2):204–13. 10.1080/00222895.2019.1602506 30982463

[18] Lucia S Aydin M Di Russo F. Sex differences in cognitive-motor dual-task training effects and in brain processing of semi-elite basketball players. Brain Sci. (2023) 13(3):443. 10.3390/brainsci13030443 36979254 PMC10046054

[19] Lucia S Bianco V Di Russo D. Specific effect of a cognitive-motor dual-task training on sport performance and brain processing associated with decision-making in semi-elite basketball players. Psychol Sport Exerc. (2023):64:102302. 10.1016/j.psychsport.2022.102302 37665802

[20] Lucia S Digno M Madinabeita I Di Russo F. Integration of cognitive-motor dual-task training in physical sessions of highly-skilled basketball players. J Sport Sci. (2024) 42(18):1695–705. 10.1080/02640414.2024.2408191 39329303

[21] Ramírez Lucas JM Párraga Montilla JA Cabrera Linares JC Latorre Román PÁ. Enhancing physical and cognitive performance in youth football: the role of specific dual-task training. J Funct Morphol Kinesiol. (2025) 10(4):404. 10.3390/jfmk10040404 41133594 PMC12551064

[22] Wollesen B Müller H Voelcker-Rehage C. Training based on multitasking – with a specific focus on motor-cognitive multitasking. In: Kiesel A Johannsen L Koch I Müller H, editors. Handbook of Human Multitasking. Berlin: Springer (2022). p. 347–97. 10.1007/978-3-031-04760-2_9

[23] Kalén A Bisagno E Musculus L Raab M Pérez-Ferreirós A Williams AM The role of domain-specific and domain-general cognitive functions and skills in sports performance: a meta-analysis. Psychol Bull. (2021) 147(12):1290–308. 10.1037/bul0000355 35404636

[24] Richlan F Weiß M Kastner P Braid J. Virtual training, real effects: a narrative review on sports performance enhancement through interventions in virtual reality. Front Psychol. (2023) 14:1240790. 10.3389/fpsyg.2023.1240790 37928573 PMC10622803

[25] Witte K Bürger D Pastel S. Sports training in virtual reality with a focus on visual perception: a systematic review. Front Sports Act Living. (2025) 7:1530948. 10.3389/fspor.2025.1530948 40181931 PMC11966202

[26] Cariati I Bonanni R Cifelli P D'Arcangelo G Padua E Annino G Virtual reality and sports performance: a systematic review of randomized controlled trials exploring balance. Front Sports Act Living. (2025) 7:1497161. 10.389/fsport.2025.149716 40365548 10.3389/fspor.2025.1497161 PMC12069346

[27] Pastel S Pauli D Bürger D Witte K. Development and testing of a virtual reality-based gait analysis tool – A pilot study. Virtual Real. (2025) 29(4):168. 10.1007/s10055-025-01248-4

[28] Bürger D Ritter Y Pastel S Sprich M Lück T Hacke M Stucke C Witte K. The impact of virtual reality training on learning gymnastic elements on a balance beam with simulated height. Int J Comput Sci Sport. (2022) 21(1):93–110. 10.2478/ijcss-2022-005

[29] Ritter Y Droste M Bürger D Pastel S Witte K. Comparison of response behavior in karate kumite between real world and virtual reality. Sports Eng. (2022) 25(1):14. 10.1007/s12283-022-00378-1

[30] Heilmann F Schubert T. The influence of specific cognitive training in virtual reality on the inhibition of professional elite young ice hockey players. Front Sports Act Living. (2025) 7:1682165. 10.3389/fspor.2025.1682165 41245651 PMC12611849

[31] Friedrich MF. Immersive training in ice hockey: evaluating the impact of virtual reality on engagement, enjoyment, and motivation. Int J Sports Sci Coach. (2025) 20(6):2551–60. 10.1177/17479541251343272

[32] Bloechle JL Audiffren J Sauthier Q Mertenat Q Waeber Y Aebischer D Perceptual training in ice hockey: bridging the eyes-puck gap using virtual reality. Sports Med. (2025) 11(1):38. 10.1186/s40798-025-00840-x PMC11993527 40220079

[33] Silva LM Neiva HP Marques MC Izquierdo M Marinho DA. Effects of warm-up, post-warm-up, and re-warm-up strategies on explosive efforts in team sports: a systematic review. Sports Med. (2018) 48(10):2285–99. 10.1007/s40279-018-0958-5 29968230

[34] Chuntonov O. Introducing the Cognitive Warm-Up: Increasing Reading Training Effectiveness for Dyslexic Individuals. [dissertation]. Haifa (Isreal): University of Haifa (2015).

[35] Emirzeoğlu M Ülger Ö. The acute effects of cognitive-based neuromuscular training and game-based training on the dynamic balance and speed performance of healthy young soccer players: a randomized controlled trial. Games Health J. (2021) 10(2):121–29. 10.1089/g4h.2020.0051 33170049

[36] Hine K Itoh Y. Warm-up cognitive activity enhances inhibitory function. PLoS One. (2018) 13(10):e0206605. 10.1371/journal.pone.0206605 30372467 PMC6205605

[37] Bouchard C Malina RM. Genetics of physiological fitness and motor performance. Exerc Sport Sci Rev. (1983) 11(1):306. 10.1249/00003677-198301000-00011 6350021

[38] Bouchard C. Genomic predictors of trainability. Exp Physiol. (2012) 97(3):347–52. 10.1113/expphysiol.2011.058735 21967902

[39] Bouchard C An P Rice T Skinner JS Wilmore JH Gagnon J Familial aggregation of Vo2max response to exercise training: results from the HERITAGE family study. J Appl Physiol. (1999) 87(3):1003–8. 10.1152/jappl.1999.87.3.1003 10484570

[40] Sparks LM. Exercise training response heterogeneity: physiological and molecular insights. Diabetologia. (2017) 60(12):2329–36. 10.1007/s00125-017-4461-6 29032385

[41] Hopkins WG. How to interpret changes in an athletic performance test. Sportscience. (2004) 8:1–7.

[42] Batterham AM Hopkins WG. Making meaningful inferences about magnitudes. Int J Sport Physiol. (2006) 1(1):50–7. 10.1123/ijspp.1.1.50 19114737

[43] Hopkins W Marshall S Batterham A Hanin J. Progressive statistics for studies in sports medicine and exercise science. Med Sci Sport Exer. (2009) 41(1):3–12. 10.1249/MSS.0b013e31818cb278 19092709

[44] Harry JR Hurwitz J Agnew C Bishop C. Statistical tests for sports science practitioners: identifying performance gains in individual athletes. J Strength Cond Res. (2024) 38(5):e264–72. 10.1519/JSC.0000000000004727 38662890

[45] Hopkins WG. Spreadsheets for analysis of controlled trials, with adjustment for a subject characteristic. Sportscience. (2006) 10:46.

[46] Chaabouni S Methnani R Al Hadabi B Al Busafi M Al Kitani M Al Jadidi K A simple field tapping test for evaluating frequency qualities of the lower limb neuromuscular system in soccer players: a validity and reliability study. Int J Environ Res Public Health. (2022) 19(7):3792. 10.3390/ijerph19073792 35409476 PMC8998105

[47] Stroop JR. Studies of interference in serial verbal reactions. J Exp Psychol. (1935) 18(6):643–62. 10.1037/h0054651

[48] Formenti D Duca M Trecroci A Ansaldi L Bonfanti L Alberti G Perceptual vision training in non-sport-specific context: effect on performance skills and cognition in young females. Sci Rep. (2019) 9(1):18671. 10.1038/s41598-019-55252-1 31822740 PMC6904471

[49] Oswald W Roth E. Der Zahlen-Verbindungs-Test (ZVT). 2., überarbeitete und erweiterte Aufl. Göttingen, Germany: Verlag (1987).

[50] Oswald WD. ZVT – Zahlen-Verbindungs-Test. 3., überarbeitete und neu normierte Aufl. Göttingen, Germany: Hogrefe (2016).

[51] Brickenkamp R Schmidt-Atzert D Liepmann D. d2-R – Aufmerksamkeits- und Konzentrationstest. Göttingen, Germany: Hogrefe (2010).

[52] Schellig D. Corsi-Block-Tapping-Test. In: Schuhfried G, editor. Wiener Testsystem. Vienna, Austria: Schuhfried GmbH (2011).

[53] Stoet G. Psytoolkit: a software package for programming psychological experiments using Linux. Behav Res Methods. (2010) 42(4):1096–104. 10.3758/BRM.42.4.1096 21139177

[54] Stoet G. Psytoolkit: a novel web-based method for running online questionnaires and reaction-time experiments. Teach Psychol. (2017) 44(1):24–31. 10.1177/0098628316677643

[55] Verbruggen F Logan GD. Response inhibition in the stop-signal paradigm. Trends Cogn Sci. (2008) 12(11):418–24. 10.1016/j.tics.2008.07.005 18799345 PMC2709177

[56] Heppe H Zentgraf K. Team handball experts outperform recreational athletes in hand and foot response inhibition: a behavioral study. Front Psychol. (2019) 10:971. 10.3389/fpsyg.2019.00971 31133925 PMC6524689

[57] Stastny P Musalek M Roczniok R Cleather D Novak D Vagner M. Testing distance characteristics and reference values for ice-hockey straight sprint speed and acceleration: a systematic review and meta-analyses. Biol Sport. (2023) 40(3):899–918. 10.5114/biolsport.2023.122479 37398950 PMC10286618

[58] Bloch H Klein C Schwarzenbrunner K. Diagnostik und Betreuung im Eishockey. Verwaltungs-Berufsgenossenschaft (2019). p. 72–3. 10.13140/RG.2.2.23066.80326

[59] Willberg C Kohler A Zentgraf K. Construct validity and applicability of a team-sport-specific change of direction test. J Hum Kinet. (2023) 85:115–26. 10.2478/hukin-2022-0115 36643841 PMC9808802

[60] Yu Z Bi G Wang W Qin Y Song Z Wu F. What are the differences between on-ice and off-ice side-cutting maneuver? A kinematic and electromyographic comparative analysis of ice hockey players. Front Bioeng Biotechnol. (2025) 13:1692676. 10.3389/fbioe.2025.1692676 41280643 PMC12634552

[61] Turner A Brazier J Bishop C Chavda S Cree J Read P. Data analysis for strength and conditioning coaches: using excel to analyze reliability, differences, and relationships. Strength Cond J. (2015) 37(1):76–83. 10.1519/SSC.0000000000000113

