# Supplementary material for: Acute effects of virtual-reality and dual-task warm-up on game-related speed paradigms in elite ice hockey athletes
Source: Front Sports Act Living. 2026 Mar 25;8:1766605. doi: 10.3389/fspor.2026.1766605 (PMC13058801; doi:10.3389/fspor.2026.1766605)
Supplement: Supplementary file 1 [file Datasheet1.pdf]

Supplementary Material

1. Change of Direction Test (CODT)
2. Lateral Shuttle (LS)
3. Dual-task paradigms
4. Dual-task training
5. Virtual-reality training

1. **Change of Direction Test (CODT)**

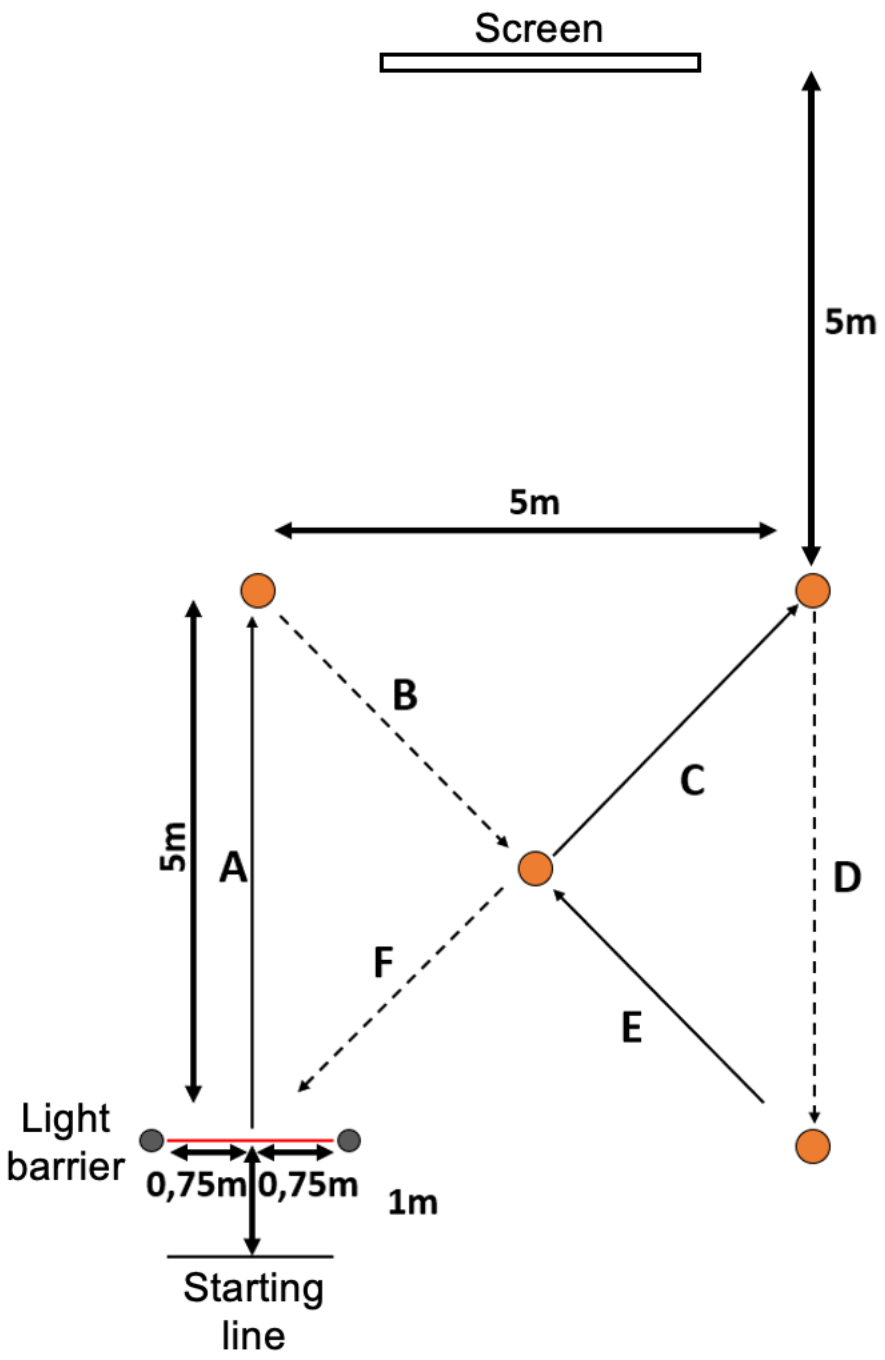

2. **Lateral Shuttle (LS)**

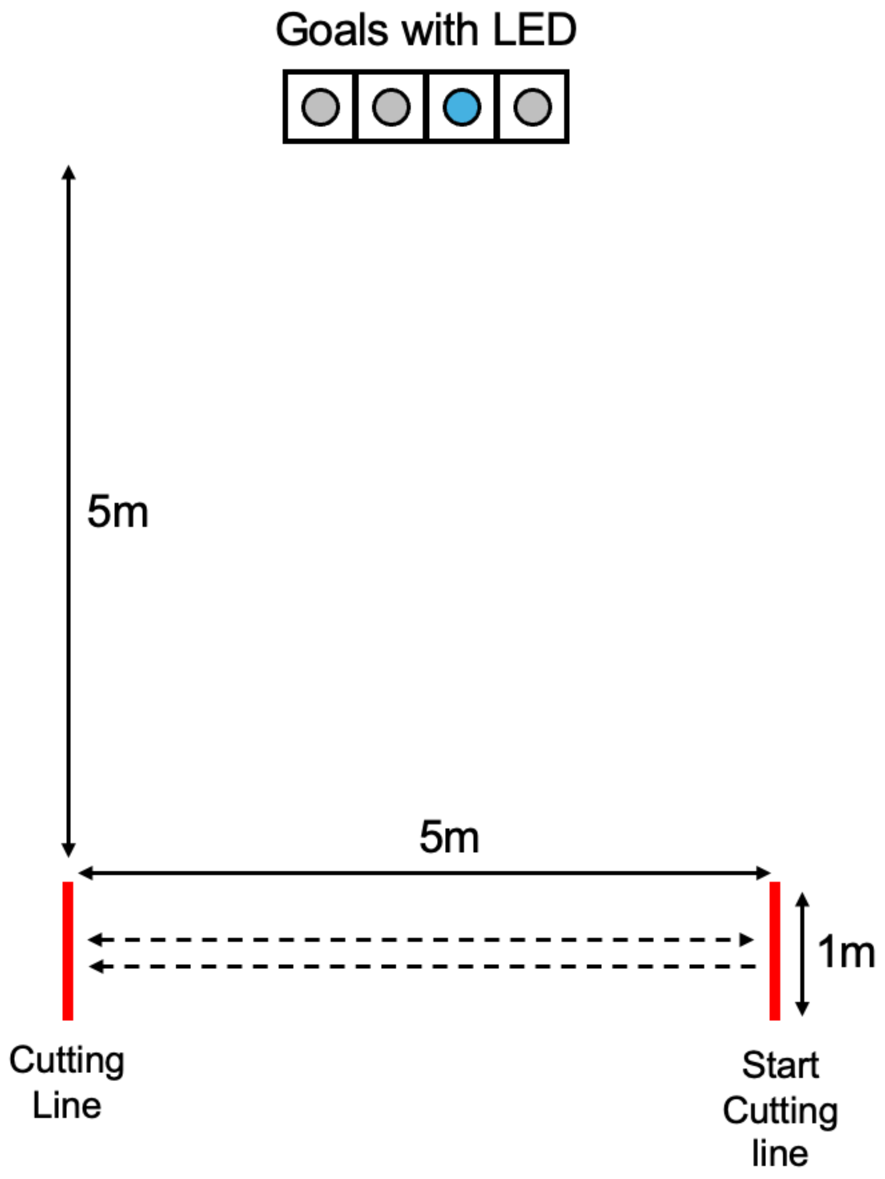

3. Setup dual-task paradigms

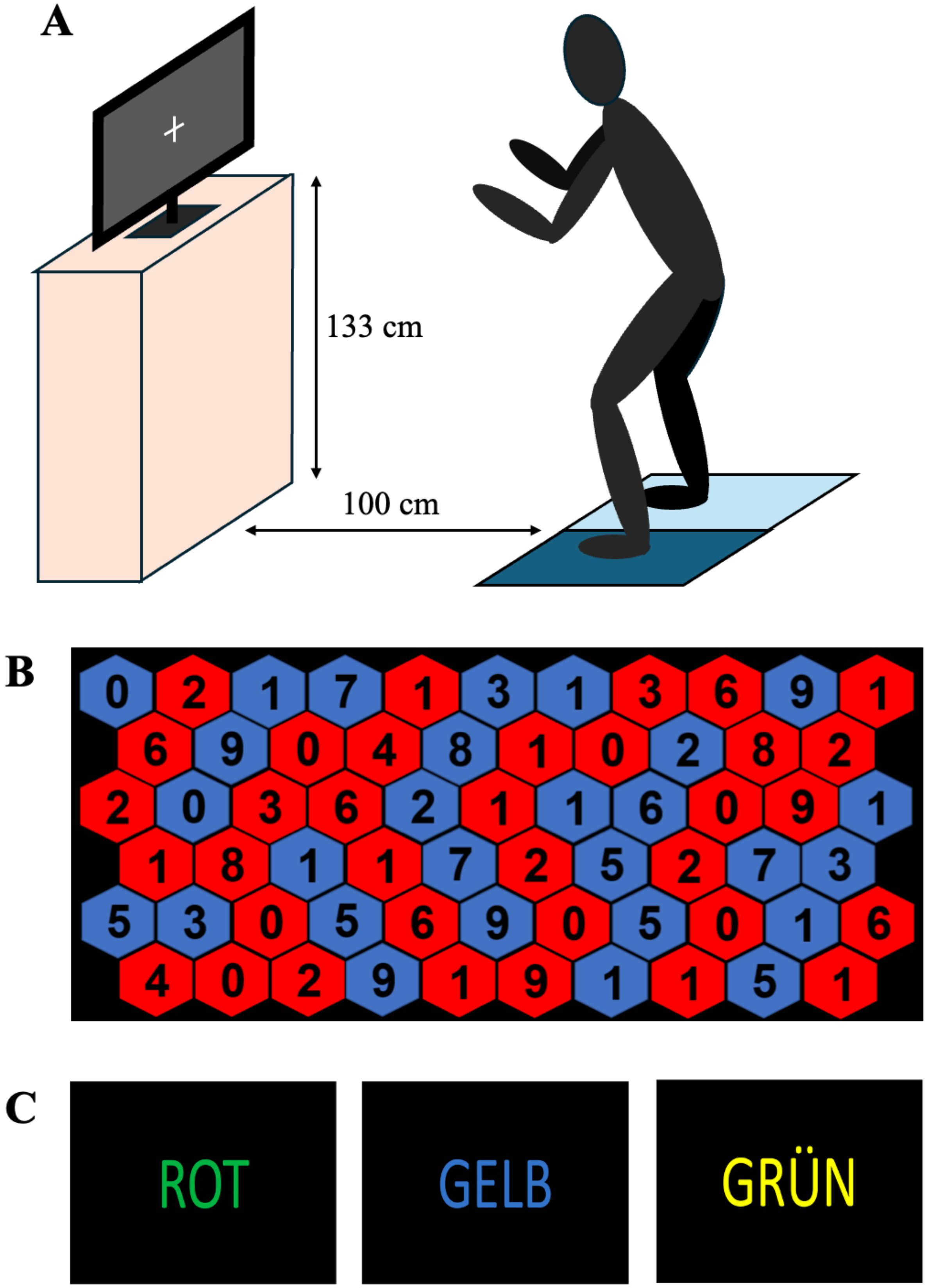

A) tapping Setup, B) exemplary SR stimulus, C) exemplary STR stimuli

4. Dual-task training

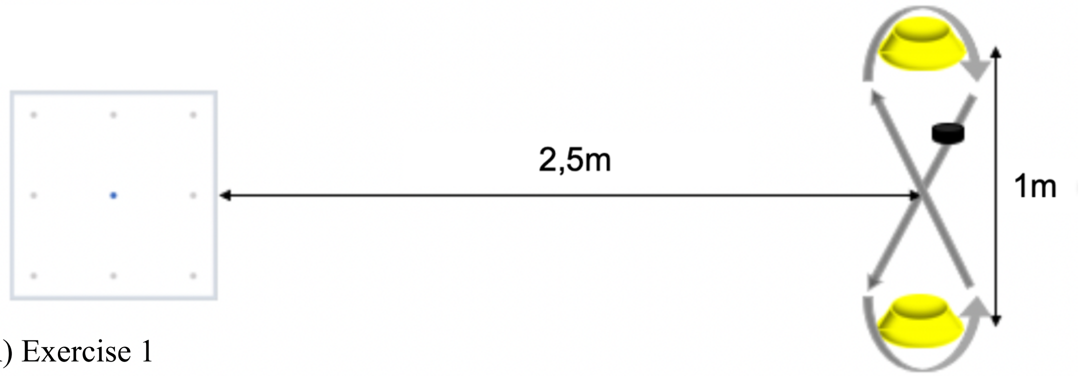

A) Exercise 1

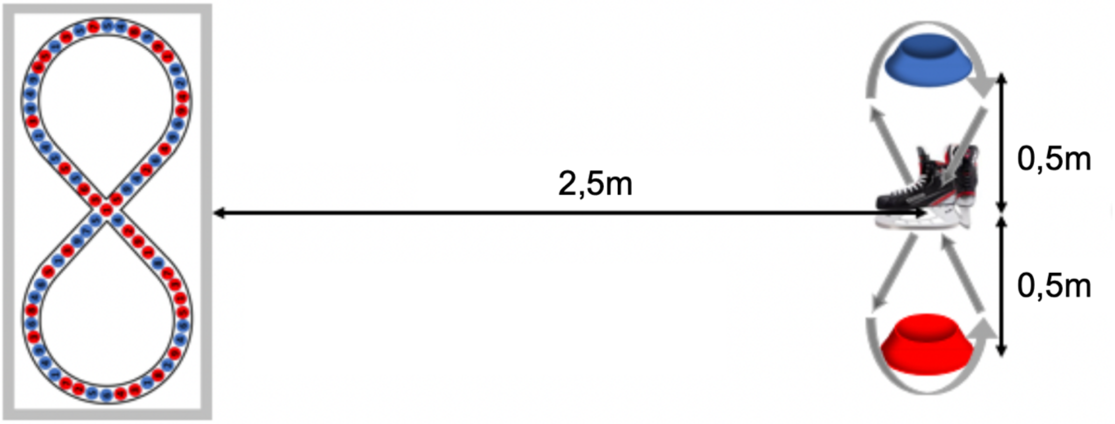

B) Exercise 2

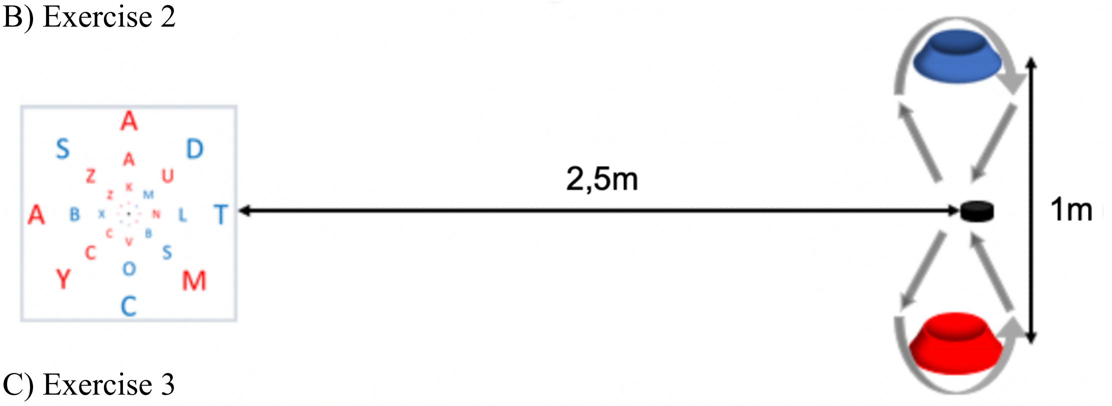

C) Exercise 3

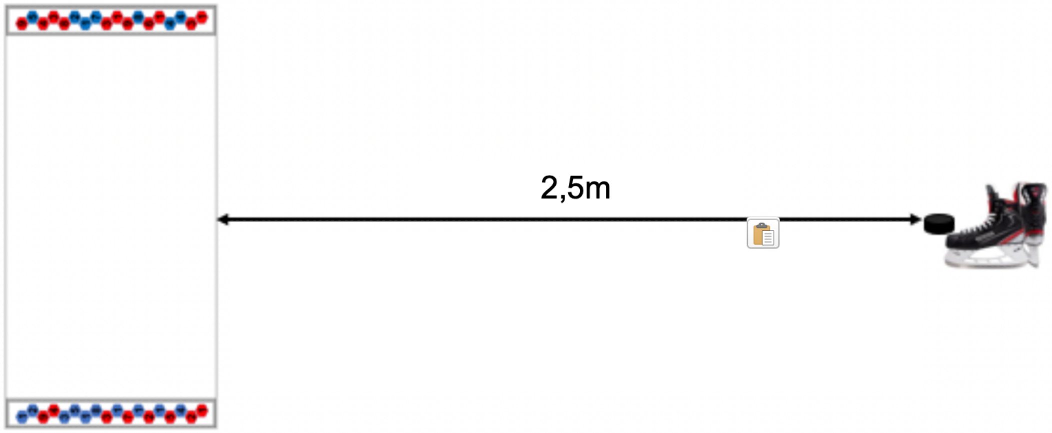

D) Exercise 4

5.Virtual-reality training setup and software

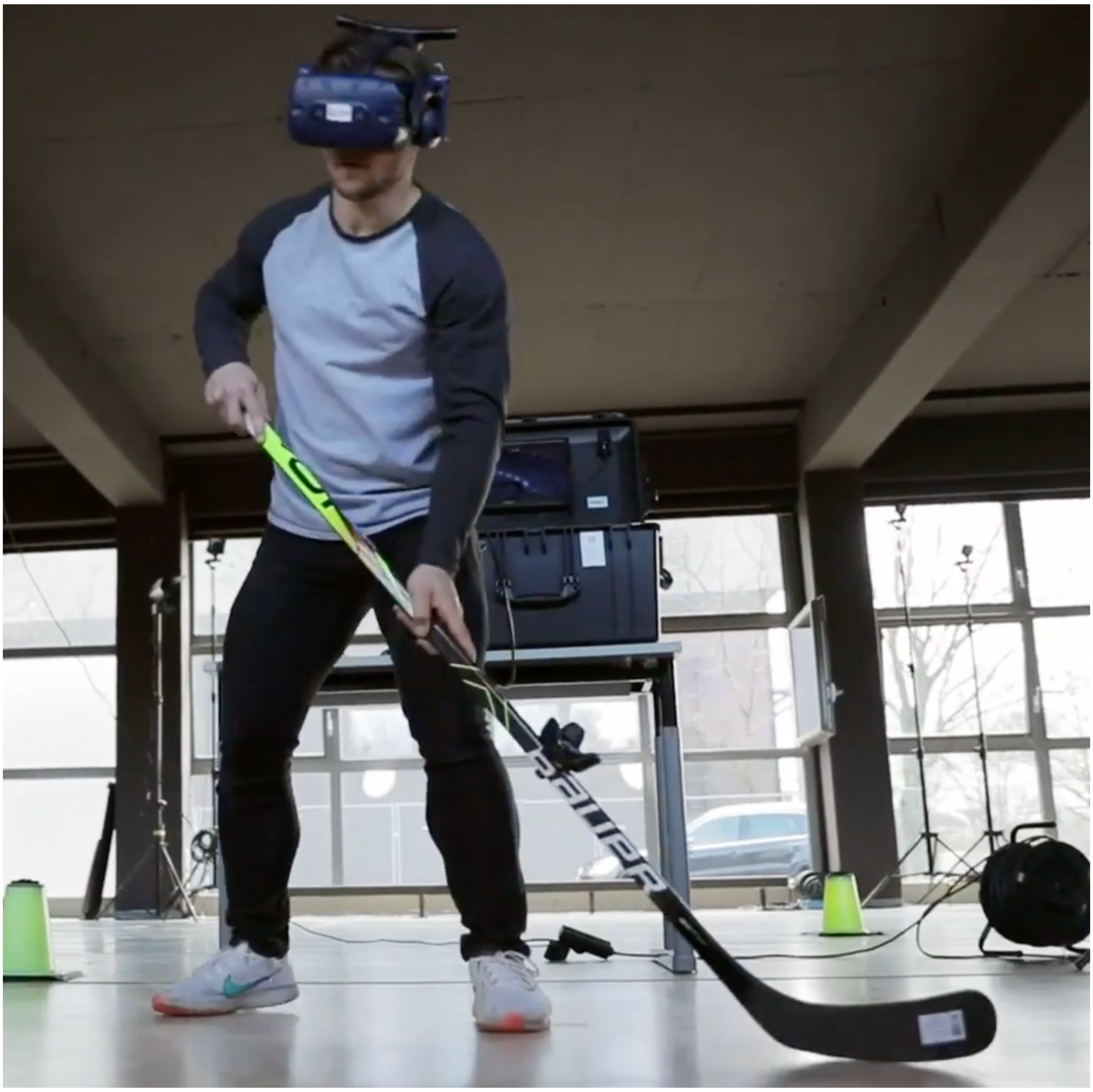

A) Setup of virtual-reality training (head-mounted device, stick with integrated sensor)

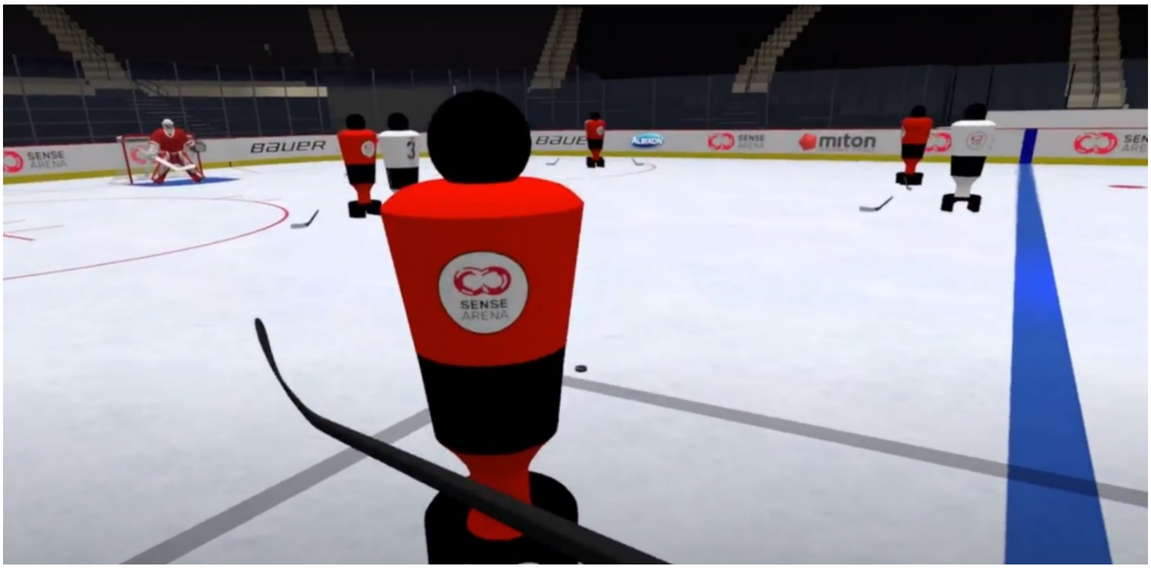

B) Exemplary first-person view of the virtual-reality software SenseArena©
